# Pediatric predictive testing to inform preimplantation genetic testing: A case report and review of the literature

**DOI:** 10.1002/jgc4.70053

**Published:** 2025-05-15

**Authors:** Kendra L. Schaa, Renata Thoeny, Rebecca J. Benson, Graeme J. Pitcher, Shelby Romoser, Alpa Sidhu

**Affiliations:** ^1^ Department of Obstetrics and Gynecology University of Iowa Health Care Iowa City Iowa USA; ^2^ Division of General Pediatrics and Adolescent Medicine, The Stead Family Department of Pediatrics, Affiliate Faculty, Program of Bioethics and Humanities University of Iowa Health Care Iowa City Iowa USA; ^3^ Division of Pediatric Surgery, Department of Surgery, Affiliate Faculty, Program of Bioethics and Humanities University of Iowa Health Care Iowa City Iowa USA; ^4^ Division of Medical Genetics and Genomics, The Stead Family Department of Pediatrics University of Iowa Health Care Iowa City Iowa USA

**Keywords:** ethics, genetic counseling, hereditary cancer predisposition, parents, predictive genetic testing, reproductive planning, shared decision making

## Abstract

Clinical genetic testing is rapidly expanding in reproductive, pediatric, and adult specialties. We report the case of a couple's request for pediatric genetic testing for a familial Lynch syndrome pathogenic variant, with the goal of utilizing this information to perform preimplantation genetic testing (PGT) on cryopreserved embryos. We outline existing professional guidelines related to genetic testing of embryos and minors for adult‐onset conditions. By highlighting conflicting perspectives from various interested parties, the significant ethical ambiguity in pediatric predictive genetic testing is underscored. This case exemplifies the value of a multidisciplinary team approach and shared decision‐making model to guide parental requests for predictive genetic testing of a minor for the purpose of PGT.

**What is known about this topic:**

Pediatric genetic testing for adult‐onset conditions is not currently recommended unless there is a clear medical benefit during childhood. There is a lack of guidance from professional organizations and other interested parties as to when this testing may be appropriate.

**What this paper adds to the topic:**

As genetic testing becomes more ubiquitous across specialties, requests for pediatric genetic testing for adult‐onset conditions are expected to increase to facilitate preimplantation genetic testing. We outline one such case, the first to our knowledge to be published, and highlight the conflicting published guidelines surrounding preimplantation, prenatal, and pediatric genetic testing for adult‐onset conditions, along with various perspectives from affected parties.

## 1 INTRODUCTION

The frequency and breadth of clinical genetic testing are rapidly expanding across pediatric and adult specialties. Simultaneously, preimplantation genetic testing for monogenic conditions (PGT‐M) is increasingly used for individuals with a personal or family history of a genetic condition (Roche et al., 2021). In 2021, in approximately 17% of in vitro fertilization (IVF) cycles in the United States, the use of at least one kind of PGT was cited as the reason for pursuing ART compared to only 4% of IVF cycles in 2010 (Centers for Disease Control and Prevention, 2023). The rise in PGT includes its growing use for adult‐onset conditions, such as tumor predisposition genes, with up to 25% of PGT‐M performed for such disorders (Besser et al., 2021; Poulton et al., 2018).

PGT‐M can be a challenging process and is not always technically feasible. PGT‐M standardly requires custom test development (referred to as “making the probe”), which often necessitates procurement of DNA samples from relatives. Genetic testing of a minor for an adult‐onset condition may be requested by the PGT lab to allow for probe development, although there are no explicit guidelines condoning this practice (Practice Committee and Genetic Counseling Professional Group of the American Society for Reproductive Medicine, 2023). Testing children for such conditions, generally, is not a well‐accepted practice among healthcare providers, thus creating a barrier for some families to use PGT‐M for their familial variant (Borry et al., 2006; Botkin et al., 2015; Ross et al., 2013).

This report describes a patient with Lynch syndrome who desired PGT‐M on her cryopreserved embryos. However, for the lab to develop the probe for her particular variant to allow for preimplantation testing of her embryos, targeted testing of her 5‐month‐old son for the familial *PMS2* variant was necessary. This case highlights clinical and ethical considerations related to genetic testing of a minor for an adult‐onset condition in the context of family planning and underscores the inconsistencies among professional guidelines and position statements related to this testing.

## 2 CASE REPORT

A 34‐year‐old nulligravida female was diagnosed with endometrial cancer. Prior to the patient's cancer treatment, she underwent oocyte retrieval and embryo creation. Preimplantation genetic testing for aneuploidy (PGT‐A) was performed on nine embryos, resulting in six euploid embryos—one male and five females. Following this testing, the patient was identified to have a de novo heterozygous *PMS2* pathogenic variant (deletion of exons 13–14) associated with Lynch syndrome. Lynch syndrome is an autosomal dominant tumor predisposition syndrome that increases the lifetime risk for colorectal, endometrial, ovarian, stomach, pancreas, urothelial tract, small bowel, and biliary tract cancer (Lynch et al., 2015).

The patient presented to genetic counseling to discuss the option of PGT‐M and prenatal diagnostic testing for the *PMS2* variant. Multiple factors were barriers to custom test development, including the inability to establish linkage due to the de novo nature of the deletion and the lack of feasibility of direct variant analysis because the specific *PMS2* breakpoints were unknown. Long‐range Sanger sequencing was pending for breakpoint mapping at the time of the patient's genetic counseling consult. Breakpoints were eventually identified, and the laboratory attempted to design a pair of primers to detect the variant by GAP‐PCR, which failed on the embryo biopsy samples after two attempts.

The patient proceeded with the use of a gestational carrier and the transfer of a male embryo with uncertain *PMS2* status, as her primary concern was the risk for endometrial cancer in a future child. She declined interest in prenatal diagnostic testing for the familial variant, citing that pregnancy termination of an affected fetus was not an option for her. This embryo transfer resulted in the birth of a healthy son. When this child was 5 months old, the patient presented for follow‐up genetic counseling to discuss the option of testing her son for the familial *PMS2* variant. If he were positive, she expressed that this information may enable custom test development, thus facilitating PGT‐M for the five remaining cryopreserved female embryos.

The subsequent genetic counseling consult with this patient and her partner focused on the couple's motivations for testing their son, as well as a review of the limitations, risks, and benefits of PGT‐M. Even if PGT‐M probe development was possible, the couple were counseled that embryo re‐biopsy may be required. Furthermore, while PGT‐M has a high accuracy rate, with studies suggesting lower than a 1% misdiagnosis rate, there remains a possibility of both false positive and false negative results (Hardy, 2020; Wilton et al., 2009). Finally, the couple were counseled that probe development would not be possible if their son tested negative, given the laboratory's need for two generations of individuals carrying the pathogenic variant. Given the complexity of this case, a clinical ethics consult was requested.

## 3 DISCUSSION

We review existing professional guidelines related to genetic testing of embryos and minors, as well as examine perspectives and ethical considerations surrounding the practice of genetic testing for adult‐onset conditions.

### 3.1 Current pediatric genetic testing guidelines

Current consensus from guidelines and position statements surrounding predictive genetic testing of minors recommends deferring any predictive testing of minors, when no changes to medical management are available or when results will not significantly benefit the child, until a patient reaches the age of legal adulthood (Borry et al., 2006; Botkin, 2016; Botkin et al., 2015; National Society of Genetic Counselors, 2018; Ross et al., 2013). The justification for this recommendation has many facets, including, but not limited to, the potential psychosocial impacts of knowing about a genetic risk factor at a young age, the risk of the child being cared for or treated differently by parents/guardians, the inability for a child to make autonomous decisions, and the child's right to an ‘open future’ (Botkin, 2016). However, subjective language is contained within these guidelines and exceptions occur. As an example, predictive testing of a minor may be considered permissible if a family is undergoing significant psychosocial distress or to facilitate specific life‐planning decisions (Botkin et al., 2015; Ross et al., 2013).

A nuance to the opposition for pediatric predictive testing is found in the American College of Medical Genetics and Genomics' (ACMG) guidelines for reporting of secondary findings (SFs) in clinical exome and genome sequencing. Genes associated with various hereditary tumor predisposition syndromes, including Lynch syndrome, are on the ‘minimum list’ of genes considered medically actionable for SF reporting. When ordering exome or genome sequencing on a minor, parents are routinely given the option to opt‐in or opt‐out of reporting SFs for their child. This is based on the guidelines which state, “the option to receive SFs should be offered regardless of the age of the patient. The best interest of the child should still be prioritized when disclosing risk for adult‐onset conditions in minors.” It was felt by this working group that the possible benefits of reporting on such conditions outweighed the potential harm to a minor (Green et al., 2013; Miller et al., 2021). Similarly, the American Society of Human Genetics recommends that “clinicians offer to disclose secondary findings for a child to the child's parents or guardians only when the information has clear clinical utility for the child and/or his or her family members” (Botkin et al., 2015).

### 3.2 Current pre‐implantation genetic testing guidelines

Pre‐implantation genetic testing for monogenic disorders (PGT‐M) is currently available for adult‐onset conditions. This testing is considered ethically permissible “when the conditions are serious and when there are no known interventions for the conditions, or the available interventions are either inadequately effective or are perceived to be significantly burdensome” (Ethics Committee of the American Society for Reproductive Medicine, 2018). The definition of “serious” or “significantly burdensome” is not universally agreed‐upon, but instead informed by an individual and family's lived experiences. For conditions which do not meet this subjective definition, PGT‐M is “ethically acceptable as a matter of reproductive liberty” based on the current scientific understanding that embryo biopsy is a relatively low‐risk procedure (Ethics Committee of the American Society for Reproductive Medicine, 2018).

Given the limitations of PGT‐M, prenatal diagnostic testing should be discussed with prospective parents as an option to confirm results or as an alternative to PGT‐M (Ethics Committee of the American Society for Reproductive Medicine, 2018). The National Society of Genetic Counselors recently revised its position statement on prenatal testing for adult‐onset conditions by removing language cautioning against this testing to acknowledge the complexities of testing for such conditions in pregnancy (National Society of Genetic Counselors, 2024). Yet, one large governing body cautions against prenatal testing for adult‐onset disorders unless the results will be used for decision‐making related to pregnancy termination or for childhood management (ACOG Committee Opinion No. 410, 2008). While a patient can voice a desire for pregnancy termination in the case of an affected pregnancy, decisions can change among prospective parents after receipt of genetic information.

### 3.3 Patient perspectives

It is well established that personal and family history will influence one's risk perception for disease, including cancer, as well as their decisions about engagement in health‐protective behaviors or screening recommendations (D'Agincourt‐Canning, 2005; Hong et al., 2020). During the genetic counseling consult with the couple, the patient expressed that one of her main concerns driving her desire for PGT‐M was the potential risk for endometrial cancer in a future daughter. She expressed grief regarding her own reproductive journey, as well as predicted guilt she would feel should her own daughter require a hysterectomy and, therefore, assisted reproductive technology for biological family building. The patient's goals of having a healthy child, preventing the future suffering of a child, and reducing future personal guilt are consistent with motivations for PGT reported in the literature (Hughes et al., 2021). These motivations may be driven by one's sense of self‐identity as a parent. A child's illness can be viewed by the parent as a failure, triggering feelings of guilt that obscure this self‐identity and influence parental decision‐making (Feudtner et al., 2018; Kon & Morrison, 2018).

In Western culture, the medical decisions for children are often considered a shared responsibility of parents and healthcare providers. When healthcare decisions are made by parents, they utilize a set of values or beliefs which fit under the theme of parents striving to be a ‘good person’ or a ‘good parent.’ This ingrained sense of duty to their child has been studied in the context of medically complex or terminal children, but is also applicable to other health care choices, in addition to certain daily decisions about parenting (Feudtner et al., 2018; Hinds et al., 2009; Neefjes, 2023). This sense of duty may sometimes be at odds with the goals of the healthcare team but should not be ignored in a shared decision‐making model.

Parents can be motivated to receive adult‐onset genetic test results for their children for a number of reasons, including the ability to pursue early intervention or surveillance, implications for family health, and the ability to prepare for a future illness, as found in one recent study (Pereira et al., 2023). These previously identified motivations are in line with the motivation felt by the patient in this case. The patient felt that knowledge of her son's Lynch syndrome status could provide the family with actionable information. A negative result would provide reassurance that their child is not at increased risk for Lynch syndrome. Even though PGT‐M could not be pursued in this case, the patient would feel that she exhausted all possible options for creating a custom probe and, in a sense, that she fulfilled her personal sense of duty. Alternatively, a positive result may allow for PGT‐M and provide the family with information they perceived to be helpful in raising their son. In particular, the couple expressed that this information could impact lifestyle and health care choices, such as encouraging their son to eat a healthier diet from early in life, which could potentially partially mitigate his risk for Lynch syndrome‐related cancers. Although no lifestyle modification can eliminate the risk for cancer in the setting of a hereditary tumor predisposition, there is literature suggesting that certain risk‐modifying behaviors, such as exercise and not smoking, can lower lifetime cancer risk (Dashti et al., 2018; Van Duijnhoven et al., 2013).

### 3.4 Ethical considerations

A driving argument against pediatric testing for adult‐onset conditions is preservation of the child's autonomy and right to an open future. Data suggests that adults who are at‐risk for a cancer predisposition syndrome, despite expressing a high interest in genetic testing, only pursue testing approximately 41% of the time (Frey et al., 2022). Therefore, it is difficult to predict whether, given the choice, this patient's child would elect to pursue familial *PMS2* variant testing or whether he would come to regret having the knowledge about his risk status as an adult.

In considering the right of a child to an open future, the potential for insurance discrimination is often introduced. While federal law offers protection from health insurance discrimination, this legislation does not apply to long‐term care, long‐term disability, and life insurance (The Genetic Information Nondiscrimination Act of 2008, 2008). The concern related to insurance discrimination can be and was mitigated by appropriate pre‐test genetic counseling that included discussing the limitations of GINA, analogous to the informed consent process with exome and genome sequencing, although it may never be eliminated completely.

Recommendations against predictive genetic testing of minors also seek to protect adolescents from adverse psychosocial impacts. Yet, evidence does not support the prevalence of this presumed harm. Two systematic reviews of young adults and adolescents who underwent pre‐symptomatic genetic testing and were identified to have a pathogenic variant found that serious adverse psychological outcomes were uncommon (Godino et al., 2016; Wakefield et al., 2016). However, most research has involved minors who were involved, to some extent, in the decision‐making process. There is a lack of literature surrounding the impact of genetic testing on children who were not involved in the decision for pre‐symptomatic genetic testing.

As stated above, there are circumstances in which children may learn predictive genetic testing information through other means, such as in the reporting of SFs from exome sequencing. Justification for disclosing SFs for a minor includes when there is clear clinical utility for the child and/or their family members and takes into account respect for parental decision‐making (Botkin et al., 2015; Green et al., 2013). In our case, the ability for custom probe development to reduce the couple's risk of having a potentially second affected child can be considered one such example of clinical utility.

Finally, some adult‐onset conditions, including Lynch syndrome, could result in significant lifetime healthcare expenses due to ongoing testing, surveillance, and/or treatment. PGT‐M could, therefore, reduce overall lifetime healthcare expenses (Ethics Committee of the American Society for Reproductive Medicine, 2018). Healthcare decision‐making is not isolated purely to the presenting patient; according to the shared model of decision‐making, providers also have responsibilities related to overall public health and societal well‐being (Opel, 2018). As such, decisions regarding the implementation of PGT‐M should incorporate the assessment of possible healthcare cost burden, when appropriate.

## 4 CONCLUSION

This case highlights the clinical and ethical considerations surrounding predictive genetic testing of a minor for the primary purpose of risk reduction in reproductive planning. Our literature review on preimplantation, prenatal, and pediatric genetic testing for adult‐onset conditions exemplifies the subjectivity and inconsistency in the application of these guidelines to our case. We illustrate the importance of nuanced discussions surrounding autonomy, legal/social implications, and personal sense of duty, with respect to and response to parental preferences, guided by genetic counseling and medical ethicists. With the expanding utilization of genetic testing, we anticipate providers will be involved in a growing number of requests for genetic testing of minors related to PGT‐M, although it is difficult to predict how current and future testing technologies, such as primary template‐directed amplification, may continue to change the landscape of testing requests. Currently, there is a lack of long‐term studies on children who have undergone genetic testing for adult‐onset conditions for the purpose of PGT in a sibling. Further research is needed in this area to inform guidelines and best practices, given the evolution of both genetic testing and societal attitudes. At the current time, we call for a case‐by‐case examination of such requests that includes eliciting, exploring, and questioning a family's values, goals, and preferences, within the framework of a shared decision‐making model of care.

## AUTHOR CONTRIBUTIONS

**Renata Thoeny:** conceptualization, writing – original draft, writing – review and editing. **Kendra L. Schaa:** conceptualization, writing – original draft, writing – review and editing. **Alpa Sidhu:** writing – review and editing. **Shelby Romoser:** writing – review and editing. **Rebecca J. Benson:** writing – review and editing. **Graeme J. Pitcher:** writing – review and editing. All authors have read and approved this submission.

## CONFLICT OF INTEREST STATEMENT

Kendra L. Schaa is the Founder of Allay Life®. Renata Thoeny, Alpa Sidhu, Shelby Romoser, Rebecca Benson, Graeme Pitcher have no conflict of interest to disclose.

## PATIENT CONSENT STATEMENT

Informed consent was obtained from this patient for the publication of this manuscript.
